# A New Approach to the Development of Disease‐Modifying Therapies for PD; Fighting Another Pandemic

**DOI:** 10.1002/mds.28310

**Published:** 2020-10-07

**Authors:** Karl Kieburtz, Russell Katz, Andrew McGarry, C. Warren Olanow

**Affiliations:** ^1^ Clintrex Research Corporation Sarasota Florida USA; ^2^ Center for Health & Technology University of Rochester Rochester New York USA; ^3^ Cooper Medical School of Rowan University Camden New Jersey USA; ^4^ Department of Neurology and Department of Neuroscience Mount Sinai School of Medicine New York New York USA

**Keywords:** Parkinson's disease, disease‐modifying therapy, regulatory approval

## Abstract

A disease‐modifying therapy that slows disease progression and development of disability is the major unmet need in the treatment of Parkinson's disease. Recent scientific advances suggest many promising and exciting new interventions. However, despite these opportunities, the cost, time and uncertainty of being able to receive an indication as a disease‐modifying therapy has caused many pharmaceutical companies to abandon development of potentially disease‐modifying drugs. We propose a new approach to development of these agents that will reduce the cost and facilitate approval of putative disease‐modifying drugs that should prove acceptable to pharmaceutical companies and regulatory agencies. © 2020 The Authors. *Movement Disorders* published by Wiley Periodicals LLC on behalf of International Parkinson and Movement Disorder Society

## 1 A Global Problem

In Jonathan Swift's satire *Gulliver*'*s Travels*, the protagonist travels to Luggnagg and encounters the Struldbrugs, individuals who are immortal but not ageless.^1^ Despite the benefits of longevity, they are subject to diseases of aging. In this novel written almost 3 centuries ago, Swift predicted our current global situation: an estimated 617 million individuals older than age 65 years and expected to grow to 2 billion by 2050.^2^ In a grim Luggnaggian scenario, modern medicine may have promoted a longer life span at the cost of greater disability because of age‐related neurodegenerative diseases.

Parkinson‘s disease (PD) is the fastest‐growing neurodegenerative disorder in terms of age‐standardized rates of disability and death^3^ and has effectively become another pandemic.^4^ It is estimated that as many as 6–10 million individuals suffer from PD globally, with an annual cost of $52 billion in the United States alone,^5^ and these numbers are likely to double within the next 30 years.^3^, ^6^ Current antiparkinsonian therapies primarily improve core motor features and do not control problems such as falling, choking, and dementia, which are the major sources of disability and institutionalization.^7^ No currently available therapy has been established to provide or approved for an indication of slowing disease progression and preventing the development of disability. In the face of this enormous public health burden, a disease‐modifying therapy that slows or prevents PD progression is the single most important unmet need in the treatment of PD.

In recent years, major genetic and other scientific advances have identified novel targets and promising candidates for a disease‐modifying intervention in PD.^8^, ^9^, ^10^, ^11^, ^12^ Targets of high interest include α‐synuclein, GBA, LRRK2, Parkin, the lysosome/autophagy system, c‐abl, and inflammation. Interventions directed at these targets are actively being studied in the laboratory, and many have demonstrated striking neuroprotective benefits in relevant animal models. Never before has the potential to develop a therapy that can slow disease progression in PD seemed more promising. However, despite these exciting advances, many pharmaceutical companies such as AstraZeneca, Bristol Meyers Squib, GSK, Pfizer, Amgen, Lilly, Sanofi, and Merck have announced they are forgoing development of “disease‐modifying” therapies for PD and even abandoning the field of CNS therapeutics altogether.^13^ Although the need is universally recognized, many companies believe that the likelihood of obtaining approval of a disease‐modifying therapy for PD is too risky for the investment required.

## 2 Obstacles

### 2.1 Time and Cost of Drug Development

According to the most recent report from the Tufts Center for the Study of Drug Development (CSDD), the cost for developing a central nervous system (CNS) drug is a staggering $2.6 billion,^14^ an 85% dollar‐adjusted increase from a mere 10 years earlier. Furthermore, the CSDD reports that compared with non‐CNS drugs, time to develop CNS drugs is 18% longer, chances of success less than 50%, time for regulatory approval 40% greater, and priority review offered only one‐third as often.^15^

### 2.2 Lack of Measurable Target Engagement

Unlike drug development for infectious diseases or oncology in which disease pathology can be directly studied and monitored (eg, biopsy, culture), in PD it is often not possible to directly measure the impact of an intervention on the intended target or on the disease process. Rather, it is frequently necessary to rely exclusively on preclinical basic science studies in deciding whether to perform long‐term and expensive clinical trials.

### 2.3 Lack of Defined Regulatory Pathway

Currently, no intervention has been approved with a “disease‐modifying” claim in PD, and it remains uncertain what clinical trial design would be acceptable to regulatory authorities.

Thus, despite the plethora of relevant targets and promising candidate disease‐modifying agents, many pharmaceutical companies are avoiding this field. Given the enormous commitment in time and resources required for drug development, they believe that a “disease‐modifying” claim is essential to justifying the risks, uncertainties, and investment. As a result, many promising interventions are not being developed at this time. It would be tragic if a viable therapy that could prevent disability is not brought forward because we lack agreement on study design and product‐labeling principles that are acceptable to both regulatory authorities and industry. It is clear that a radical change is required.

## 3 Previous Attempts to Develop a Disease‐Modifying Therapy for PD

Multiple study designs have been used to assess potential disease‐modifying therapies for PD (see Table 1).^16^, ^17^, ^18^, ^19^, ^20^, ^21^, ^22^, ^23^, ^24^, ^25^, ^26^, ^27^, ^28^, ^29^, ^30^, ^31^, ^32^, ^33^ The first major clinical trial seeking to slow PD progression was the DATATOP study.^16^, ^17^ The primary end point was the time to develop a milestone of disease progression (need for levodopa therapy). The study was robustly positive for selegiline; however, it could not be definitively determined that the observed benefit was due to a disease‐modifying effect because of a confounding symptomatic effect. Numerous study designs have tried to overcome this problem. Washout designs attempted to determine if benefits seen with an intervention persist following drug withdrawal.^18^, ^19^ However, the possibility of a long‐duration symptomatic benefit following washout could not be excluded. Change in motor progression has been used as an index of disease progression in several trials^20^, ^21^, ^22^; however, reduced deterioration in UPDRS score could clearly result from a symptomatic effect. Slowing of UPDRS progression in the practically defined OFF state has been used based on the assumption that this end point provides a better measure of the underlying disease state, but these studies have generally failed,^23^, ^24^, ^25^ and positive results could be because of long‐duration symptomatic effects rather than an effect on the disease process.^26^ Quality‐of‐life measures have been employed as a primary end point,^27^ but these assessments are subjective, potentially confounded by personal and social factors and do not necessarily correlate with clinical or biological deterioration. Surrogate imaging measures of dopamine function have been used to assess if an agent slows the rate of biomarker decline as an indication of disease modification.^28^, ^29^ Although some studies were positive, results could have been confounded by direct pharmacologic effects of the interventions on the biomarker. Novel, long‐term “simple” studies have used a global measure as a primary outcome that includes UPDRS score, falling, cognitive function, and cumulative disability.^30^ In this design, participants in mid‐stage disease who are on antiparkinsonian therapy are randomized to study drug or placebo and followed for years. During this time, physicians can treat patients with any agent they deem clinically appropriate. Although potentially providing important and useful information, this design does not provide definitive evidence that an intervention has changed the underlying disease process. Thus, none of the above‐described clinical trial designs has been sufficient for regulatory agencies to grant a disease‐modifying indication, even with positive results on the primary outcome measure.

**TABLE 1:** Examples of different clinical trial methods used to try to define disease modification in PD

Design	End point	Agent	Result	References
Time to milestone of disease progression	Time to need levodopa	Deprenyl	Positive	^15^, ^16^
		Vitamin E	Negative	
Washout design	Δ UPDRS	Selegiline	Positive	^17^
		l‐Dopa	Positive	^18^
Slow UPDRS progression	Δ UPDRS	Co‐Q10	Negative	
	Δ UPDRS	TCH‐346	Negative	^20^
	Δ UPDRS	Pioglitazone	Negative	^21^
Slow UPDRS progression	Δ UPDRS	Transplant	Negative	^22^
In practically defined off	Δ UPDRS	Neurturin	Negative	^23^
	Δ UPDRS	GDNF	Negative	^24^
	Δ MDS‐UPDRS.	Exenatide	Positive	^25^
Quality of life	Global rating	Transplant	Negative.	^26^
DA imaging	Δ FD‐PET	Ropinirole	Positive	^27^
	Δ β‐CIT	Pramipexole.	Positive	^28^
Long‐term simple study	Global statistic	Creatinine	Negative	^29^
Two‐period design	1. UPDRS — period one	Rasagiline	Positive (1 mg)	^30^, ^31^
	2. Separation early/delayed start		Negative (2 mg).	
	3. Noninferiority slope — period two			

The “delayed‐start” study has attracted attention because regulatory guidance documents suggest that positive results with this design might be acceptable for providing a disease‐modifying indication.^31^, ^32^ This design aims to determine if early treatment with an agent provides benefits that cannot be achieved with delayed treatment using the same agent.^33^ It is a complex design that requires multiple primary end points that typically include at a minimum (1) evidence that the early‐start group is superior to the delayed‐start group at the end of the study despite patients in both groups being on the same treatment and (2) evidence of noninferiority in the rate of deterioration of UPDRS slopes of the early‐ and delayed‐start groups (ie, the UPDRS scores are not converging at the end of the study, implying that early treatment provides a benefit that cannot be achieved with delayed treatment. Positive results with both these end points are consistent with an intervention having a disease‐modifying effect and are not readily explained by a symptomatic effect. However, there are multiple challenges to employing a delayed‐start design. These include determining the duration of the 2 treatment periods, minimizing dropout to ensure that the benefits of randomization are preserved when entering the second period of the study, the need to meet multiple primary end points, and prespecified agreement on the noninferiority margins of UPDRS slopes. Given these complexities, even a well‐conducted delayed‐start study can be difficult to interpret. For example, in the ADAGIO study the 1‐mg dose of rasagiline met all 3 prespecified primary end points.^34^ However, the 2‐mg dose failed and there was debate about the validity of the noninferiority margins, even though they had been prespecified. For these reasons, an application for a disease‐modifying indication was denied, even though results were positive.

Thus, there is uncertainty about which study design should be used to establish that an intervention slows clinical progression in PD.

## 4 A Possible Way Forward

We believe that a 2‐pronged approach could help to resolve this situation. First, regarding design, a simple double‐blind, placebo‐controlled, parallel‐group trial using change in UPDRS score or in on time without troublesome dyskinesia as an endponit is a well‐accepted approach to drug approval in PD. Such a study could also be designed to demonstrate the effect of an intervention on the functional impact of PD and to assess PD features not adequately helped with current therapies (eg, falling, dementia). Although this type of study would not lead to a “disease‐modifying” indication, it would provide a straightforward, rapid and established way to obtain approval as a treatment for PD, and avoids the expense, duration, complexities, and uncertainties of a delayed‐start study.

Second, rather than using terms such as “disease modification” or “neuroprotection,” a simple and clear description of relevant basic science and specific clinical findings could be described in the product label (eg, sections 12 and 14 and possibly the Highlights sections, as per current U.S. Food and Drug Administration [FDA] guidance on product labeling), as in fields such as infectious disease and cancer.^35^, ^36^, ^37^, ^38^ Controversy exists in how to define an intervention that slows, stops, or reverses PD clinical progression and the development of cumulative disability. The term “neuroprotection” seems inappropriate, as “protection” of neurons cannot be established during life. Furthermore, preservation of other cellular elements (eg, glia) or inhibiting damaging processes (eg, inflammation) may also be relevant to preserving function. The term “disease modification” was proposed as an alternative, but here, too, one cannot currently establish that a therapy actually “modifies” the disease process. These terms have not been acceptable to regulatory agencies to employ in labeling, as they are based on inference rather than empirical data.

An alternate approach is to simply report what was found in the clinical trial(s) and the relevant basic science findings in the label but not as an indication.

In the United States, clinical findings observed in an adequate and well‐controlled clinical trial can be described in section 14 of the label. These could indicate an intervention‘s effects on measures of clinical progression such as the rate of UPDRS progression and time to development of a milestone of disease progression (eg, falling, dementia, cumulative disability). It should be noted that such a beneficial effect could occur in the absence of modification of the underlying disease process per se and thus would not permit an indication as a disease‐modifying agent; however, slowing the *clinical* progression of factors listed above is a highly desirable outcome regardless of the explanation. For example, language in the label could indicate that a given intervention is associated with “less worsening of PD features (as measured by UPDRS) over 18 months,” or that “fewer individuals developing falls or cognitive impairment in 3 years.” These are quantitative descriptions of observations that can be substantiated by clinical data that incorporate clinical findings and a temporal profile and could be included in the appropriate section of the label.

The label could also include language describing relevant basic science, particularly as it applies to the mechanism of action (MOA) of the drug (included in section 12.1 of the FDA label). For example, a specific intervention could be described as preventing α‐synuclein‐based pathology, neurodegeneration, and behavioral effects in relevant animal models, while not directly influencing the dopamine system. This information is useful for prescribers because it may be germane in their interpretiation of the mechanism responsible for the observed clinical benefit.

It is obviously important to discuss with regulatory agencies in advance the potential language for a future label. European Medicines Agency guidance on neuroprotective therapies for PD,^39^ and FDA guidance on Alzheimer‘s disease^31^ can help to clarify what can be included in the label and the specific language that might be used to describe the effects of a drug on disease progression without using the terms *neuroprotection* or *disease modification*.

Despite not being likely to provide a disease‐modifying indication, there are several advantages to this approach. The trial design provides a relatively rapid, inexpensive, and well‐established pathway for regulatory approval. Precise, descriptive (not inferential) language can be used to describe the observed clinical effects (including the temporal profile) of the intervention can be included in the clinical trials section of the product label. In addition, information about an intervention‘s known MOA can also be included in the science section of product label, particularly if it is relevant to clinical effects. The inclusion of this language in the label, even without a disease‐modifying indication, permits a novel therapeutic agent to be distinguished from currently available therapies.

## 5 A 21st‐Century Call to Action

PD represent a global health crisis and a “silent” pandemic. As the global population ages, treatments to delay and lessen disability are essential to avoid a Luggnaggian outcome. New, exciting approaches that offer the possibility of slowing progression in PD exist, but the costs and uncertainties involved in testing these agents are currently prohibitive. Although the approach we propose does not assure a disease‐modifying indication, it uses a relatively inexpensive, rapid, and established drug development pathway. The inclusion of the relevant scientific data and specific clinical findings within the label could differentiate a putative disease‐modifying agent from existing therapies by showing that it acts on a mechanism implicated in the etiopathogenesis of PD and prevents the development of disabilities that are not prevented with existing therapies. And because the information is in the label, albeit not as an indication, it can be used in educational and promotional activities of drug developers and shared with physicians who can decide for themselves how to best utilize the therapy. We believe that this approach will prove acceptable to both industry and regulators, and will facilitate the development of therapies that have the potential to forestall disability for patients with PD.

## Author Roles

K.K: conception, writing first draft, review and critique.

R.K.: review and critique.

A.M.: review and critique.

C.W.O.: writing first draft, review and critique.

## Conflicts of Interest (Past 12 Months)

K. Kieburtz: consultant for Clintrex Research Corp., Roche/Genentech, Novartis, Blackfynn LLC; grant support from NIH (NINDS, NCATS), Michael J. Fox Foundation; ownership in Clintrex Research Corp., Hoover Brown LLC, Safe Therapeutics LLC. R. Katz R: consultant for Clintrex. A. McGarry: consultant for Clintrex. C. W. Olanow: Shares in Clintrex Research Corporation and Neuro Research Corporation.
